# Myeloid-derived suppressor cells in multiple myeloma: mechanistic insights and therapeutic implications

**DOI:** 10.3389/fimmu.2026.1815669

**Published:** 2026-09-15

**Authors:** Jingjing Wen, Qiaolin Zhou, Jing Yue, Ya Zhang, Yuhuan Zheng, Fang Xu

**Affiliations:** 1 Department of Hematology, Mianyang Central Hospital, School of Medicine, University of Electronic Science and Technology of China, National Health Commission Key Laboratory of Nuclear Technology Medical Transformation, Mianyang, Sichuan, China; 2 Department of Hematology/Institute of Hematology, West China Hospital, Sichuan University, Chengdu, Sichuan, China

**Keywords:** immunosuppression, immunotherapy, multiple myeloma, myeloid-derived suppressor cells, targeted therapy, tumor microenvironment

## Abstract

Multiple myeloma (MM) is an incurable malignancy and ranks as the second most common hematological cancer in western countries. Relapsed disease is common in MM patients, and is largely influenced by the establishment of an immunosuppressive tumor microenvironment (TME) and concomitant systemic immune impairment. Myeloid-derived suppressor cells (MDSCs) are crucial regulators of immune homeostasis and response in various pathological states, including cancer. Within the TME, MDSCs and tumor cells engage in bidirectional crosstalk through both direct and indirect mechanisms, collectively driving immune evasion and tumor progression. As a principal immunosuppressive population within the MM milieu, MDSCs play a central role in the immunoregulation of MM. Although immunotherapy has emerged as a pivotal treatment modality for MM, MDSCs, the key constituents of the TME, constitute a major source of resistance to such therapies. This review elucidates the mechanisms governing MDSCs interactions within the myeloma TME, discusses potential therapeutic strategies targeting MDSCs, and outlines a rational framework for integrating MDSC-directed approaches with current MM therapies and immunotherapy.

## 1 Introduction

Multiple myeloma (MM) is the most common hematologic malignancy in terms of both incidence and mortality (1). It is driven by the clonal expansion of malignant plasma cells in the bone marrow (BM) and remains incurable due to inevitable relapse (2). Disease progression is not solely determined by tumor-intrinsic alterations but is critically orchestrated by a profoundly immunosuppressive tumor microenvironment (TME) (3, 4). This permissive niche, shaped by cellular components (e.g., stromal cells, myeloid cells) and soluble factors, not only supports myeloma cells survival but also actively undermines therapeutic efficacy (4, 5).

Within this immunosuppressive landscape, myeloid-derived suppressor cells (MDSCs) have emerged as pivotal regulators. MDSCs are a heterogeneous population of immature myeloid cells that expand under pathological conditions, including cancer, and are recruited to tumor sites where they exert potent immunosuppressive functions, facilitating immune evasion and disease progression (**Figure 1**) (6–8). In MM, MDSCs are a critical component of the TME and play a major role in establishing and maintaining the immunosuppressive niche (9, 10). Consequently, targeting MDSCs represents a promising strategy to overcome microenvironment-mediated resistance and improve outcomes in MM (11, 12).

**Figure 1:**
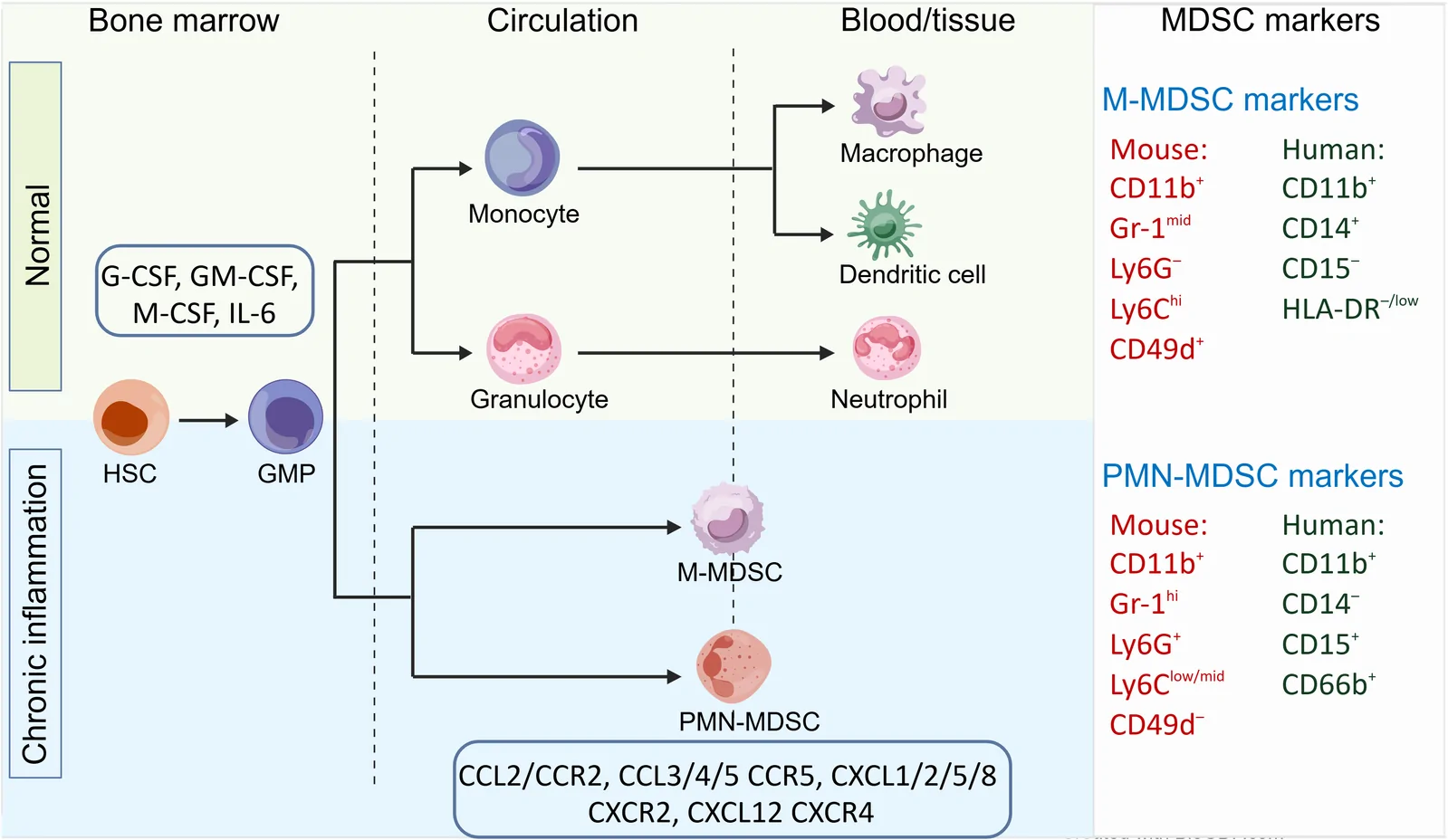
Schematic of myeloid-derived suppressor cell differentiation and recruitment. Hematopoietic stem cells (HSCs) differentiate in the bone marrow into granulocyte-monocyte progenitors (GMPs), which further develop through the hematopoietic system. Under normal conditions, bone marrow-derived myeloid cells (HSCs and GMPs) develop into cells of the innate immune system, including macrophages, dendritic cells, and neutrophils. However, in the context of chronic inflammation associated with advanced cancer, inflammatory cytokines such as G-CSF, GM-CSF, M-CSF, and IL-6 promote the proliferation and differentiation of myeloid-derived suppressor cells (MDSCs), which include monocytic MDSCs (M-MDSCs) and polymorphonuclear MDSCs (PMN-MDSCs). These cells migrate into the bloodstream and tissues, including the tumor microenvironment (TME). Within the TME, MDSCs produce immunosuppressive cytokines that shield tumors from the host immune system and immunotherapy. Furthermore, proinflammatory chemokine such as CCL2/CCR2, CCL3/4/5-CCR5, CXCL1/2/5/8-CXCR2, and CXCL12-CXCR4 are pathologically activated and contribute to recruitment of M-MDSCs and PMN-MDSCs. Created with BioGDP.com.

This review systematically examines the biology and therapeutic targeting of MDSCs in MM. We outline their phenotypic features, prognostic relevance, recruitment mechanisms, and bidirectional interactions with myeloma cells. Furthermore, we evaluate emerging strategies to deplete, differentiate, or functionally inhibit MDSCs, and assess their potential synergy with existing immunotherapies and standard treatments. By linking mechanistic insights to clinical translation, this review aims to provide a roadmap for leveraging MDSC-directed strategies to improve outcomes in MM.

## 2 Prognostic significance of MDSCs in myeloma

### 2.1 Phenotypic characterization

Phenotypic identification of MDSCs faces fundamental challenges, as their surface markers substantially overlap with those of conventional neutrophils and monocytes, and no single exclusive marker exists (13). MDSCs are broadly classified into two major subsets: polymorphonuclear (PMN-MDSCs) and monocytic (M-MDSCs), based on their lineage origins (**Figure 1**) (13). In mice, MDSCs are conventionally defined as CD11b^+^Gr-1^+^ cells (14, 15), with PMN-MDSCs further distinguished as Ly6G^+^Ly6C^low^/^mid^ and M-MDSCs as Ly6G^-^Ly6C^high^ (16). In humans, M-MDSCs are typically defined as CD11b^+^CD14^+^HLA-DR^-^/^low^CD15^-^, whereas PMN-MDSCs are identified as CD11b^+^CD14^-^CD15^+^ or CD66b^+^ (14, 17). PMN-MDSCs share the CD11b^+^CD14^-^CD15^+^/CD66b^+^ phenotype with neutrophils and are currently distinguished primarily by density gradient (low-density versus high-density) combined with emerging markers such as LOX-1 and CD84 (13, 18). M-MDSCs can be differentiated from peripheral blood (PB) monocytes by assessing MHC class II/HLA-DR expression levels (low/negative versus positive); however, these criteria alone may be insufficient for complete discrimination (18, 19). Recently identified molecules, including CXCR1^+^ and CD84^+^, may further refine this distinction (13, 19, 20). Furthermore, although functional immunosuppressive assays are considered the gold standard for MDSCs identification, their in vitro application carries inherent limitations (13, 21).

While these surface markers provide a foundational framework, their specificity and functional correlates in the MM microenvironment remain areas of active investigation. Emerging single-cell transcriptomic studies have begun to reveal greater heterogeneity within MDSCs populations than previously recognized based on surface markers alone, and the application of spatial transcriptomics may further elucidate the topographical distribution and niche-specific functional programs of MDSCs subsets within the MM bone marrow microenvironment. 

### 2.2 M-MDSCs in myeloma

Multiple studies have demonstrated that patients with MM exhibit significantly elevated levels of M-MDSCs in peripheral blood at diagnosis and relapse compared to healthy controls (22–25). These levels correlate positively with disease stage (22, 23). A recent study further confirmed that PB M-MDSCs levels are substantially higher in patients with progressive disease than in those at diagnosis or in complete response (26). Moreover, patients who relapsed or were resistant to induction therapy, the frequency of Arg-1^+^ and PD-L1^+^ M-MDSCs was elevated, a change likely to reinforce tumor immunosuppression (27). Following treatment, M-MDSCs levels are significantly reduced, reflecting their dynamic association with disease activity (24, 25). Clinically, M-MDSCs levels hold prognostic significance. The PB M-MDSCs count a t diagnosis has been identified as an independent risk factor for overall survival (OS) in MM patients (26). Additionally, lower pre-transplant M-MDSCs levels are associated with longer progression-free survival following autologous hematopoietic stem cell transplantation (28).

### 2.3 PMN-MDSCs in myeloma

Recent studies have shown that patients with active MM exhibit significantly higher levels of PMN-MDSCs in both BM and PB than individuals with monoclonal gammopathy of undetermined significance (MGUS), smoldering MM (SMM), or healthy controls (29–31). Patients with relapsed/refractory multiple myeloma (RRMM) exhibit a higher proportion of PMN-MDSCs in PB compared to newly diagnosed patients (32). Following treatment, PMN-MDSCs levels generally decline (25). However, a transient increase has been observed during remission after induction therapy and after the first cycle of lenalidomide-dexamethasone in RRMM (27, 33). PMN-MDSCs may also contribute to therapy resistance (32). These findings underscore the dynamic and treatment-responsive nature of PMN-MDSCs in MM. Clinically, elevated PMN-MDSCs levels in PB and BM are significantly associated with shorter OS in MM patients (31, 34). These underscoring their prognostic relevance and potential as a therapeutic target.

The dominant expansion of a particular MDSCs subset is influenced by various factors within the TME. Accordingly, the ratio of tumor-infiltrating M-MDSCs to PMN-MDSCs differs across cancer types and stages of disease progression (35, 36).

Collectively, longitudinal studies have recently elucidated the dynamic relationship between MDSCs levels and clinical outcomes in MM across different treatment settings. In a cohort of 99 newly diagnosed MM patients, VRd induction therapy significantly expanded circulating M-MDSCs, with greater expansion correlating with poorer response depth (PR vs VGPR, P = 0.0083); however, this association did not retain independent predictive value after adjustment for R2-ISS stage (20, 37). In patients undergoing ASCT, higher early MDSCs levels at engraftment were independently associated with inferior PFS (HR = 3.06, P = 0.03) (27), while elevated pre-transplant M-MDSCs correlated with shorter time to progression, with CSF1R identified as a potential target (28). Moreover, elevated M-MDSCs levels in PB at diagnosis have been identified as an independent adverse prognostic marker for OS in multivariate analysis (26). Single-cell RNA sequencing data further identified PTGS1 as a key PMN-MDSCs related gene that inhibits CAR-T cytotoxicity and correlates with poor prognosis (AUC = 0.94) (34) (**Table 1**). These findings underscore the prognostic relevance of MDSCs across treatment settings and support their potential as predictive biomarkers and therapeutic targets in MM.

**Table 1:** Prognostic value of MDSCs in MM: summary of clinical studies.

Patient number[study]	Sample type/MDSCs phenotype	Treatment status	Association with clinical outcomes
26 MM patients, 5 controls (25)	PB: PMN-MDSCs (CD11b^+^CD33^+^CD15^+^), M-MDSCs (CD11b^+^CD33^+^CD14^+^HLA-DR^-^/^low^)	Mixed (newly diagnosed, remission, relapse)	Elevated in MM vs controls; PMN-MDSCs positively associated with light chain subtype (*p* = 0.004); levels decreased after treatment.
99 NDMM patients (37)	PB: M-MDSCs (CD11b^+^CD33^+^CD14^+^HLA-DR^-^/^low^)	VRd induction → ASCT	VRd expanded M-MDSCs; greater expansion correlated with poorer response (PR vs VGPR, *p* = 0.0083); not independently predictive after R2-ISS adjustment.
MM patients undergoing ASCT (28)	PB: M-MDSCs (pre-transplant)	Pre-ASCT	Higher pre-transplant M-MDSCs correlated with shorter time to progression; CSF1R identified as a potential target.
Plasma cell myeloma patients (26)	WB: M-MDSCs	At diagnosis	Elevated PB M-MDSCs levels independently predicted inferior OS in multivariate analysis.
713 MM patients (RNA-seq), 7 CAR-T patients (scRNA-seq) (34)	BM/PB, scRNA-seq: PMN-MDSCs gene signatures (PTGS1, TLR7, PDE2A, ACTN1, CCND1)	Pre- and post-CAR-T	PMN-MDSCs risk model showed high prognostic accuracy (AUC = 0.94); PTGS1 identified as key marker associated with poor CAR-T outcomes

PB, peripheral blood; WB, whole blood; BM, bone marrow; MM, multiple myeloma; NDMM, newly diagnosed multiple myeloma; VRd, bortezomib + lenalidomide + dexamethasone; ASCT, autologous stem cell transplantation; OS, overall survival; R2-ISS, Revised International Staging System (second revision); CAR‑T, chimeric antigen receptor T‑cell; PMN‑MDSCs, polymorphonuclear MDSCs; M-MDSCs, monocytic MDSCs; AUC, area under the curve.

## 3 Recruitment and differentiation of MDSCs

The recruitment and differentiation of MDSCs within the TME is a highly complex and dynamic process. During tumor progression, three key events govern MDSCs activity: disruption of BM hematopoiesis, migration of MDSCs to the tumor site, and their subsequent local differentiation (7, 13). Impaired BM hematopoiesis promotes the generation of MDSCs in the BM, after which these cells are recruited to the tumor site by factors secreted by both tumor cells and stromal cells (38, 39).

### 3.1 Recruitment of MDSCs

The recruitment of MDSCs into the TME is primarily directed by conserved chemokine signaling axes that also guide granulocyte and monocyte trafficking (40). These include key pathways such as CCL2/CCR2, CCL3/4/5-CCR5, CXCL1/2/5/8-CXCR2, and CXCL12-CXCR4, which have been broadly implicated in MDSCs homing across various cancers (40–49).

Research on the role of these cytokines in MDSCs in MM remains limited. However, the cytokines are closely linked to the pathogenesis and progression of MM. Several cytokines showed altered levels in patients with MM and specific chemokine-receptor axes mediate the homing of myeloma cells to defined BM microenvironments (50–52). CCL2 (also known as monocyte chemoattractant protein-1, MCP-1), upregulated via macrophage–MM cell interactions, promoting an M2-like macrophage phenotype that protects tumor cells from drug-induced apoptosis and contributes to drug resistance in MM (53, 54). CCL3 (MIP-1α) promotes chemoresistance in MM via CCR1-dependent ERK/Akt/mTOR signaling and drives bone destruction by enhancing osteoclast differentiation and RANKL expression in osteocytes (55–57). CCL5 secreted by MM cells can promote the differentiation of PB mononuclear cells toward MDSCs (58). CXCL1, CXCL5, CXCL7, CXCL8, and CX3CL1 are also dysregulated in MM. Those chemokines contribute to MM proliferation, bone destruction, angiogenesis, and therapy resistance through receptors such as CXCR2 and CX3CR1, further shaping the immunosuppressive and pro-tumorigenic microenvironment (59–64). CXCL12 (also known as stromal cell-derived factor- 1, SDF-1), abundantly produced by MM bone marrow mesenchymal stem cell (BMSC) (65). The CXCL12/CXCR4 axis enhances MM cell adhesion and promotes bortezomib resistance, while also facilitating intercellular mitochondrial transfer and niche remodeling to drive disease progression (66–68). CXCL13 is elevated in the PB and BM samples of MM patients and promotes MM progression by enhancing M2 macrophage infiltration (69). BMSC-derived CXCL13 also drives tumor invasion, proliferation, and bortezomib resistance via CXCR5-mediated BTK/NF-κB/BCL-2 signaling (70).

Collectively, MM-associated chemokines not only directly support tumor survival, bone homing, and drug resistance but also establish an immunosuppressive microenvironment conducive to MDSCs recruitment and function.

### 3.2 Differentiation of MDSCs

#### 3.2.1 Molecular basis of MDSCs differentiation

The differentiation of MDSCs is governed by factors central to normal myelopoiesis, including granulocyte-macrophage colony-stimulating factor (GM-CSF), granulocyte colony-stimulating factor (G-CSF), and macrophage colony-stimulating factor (M-CSF) (71, 72). In vitro, combined stimulation with GM-CSF and interleukin-6 (IL-6) can direct murine myeloid precursors toward an immunosuppressive MDSCs phenotype (73). Within the TME, these cytokines—secreted by both tumor and stromal cells—drive the differentiation of hematopoietic progenitors into MDSCs primarily through activation of the STAT3 and STAT5 signaling pathways (38, 74, 75). In MM, malignant plasma cells and bone marrow stromal cells are major producers of key cytokines such as IL-6 and GM-CSF (76–78). These cytokines help establish a permissive niche for MDSCs generation and contributing to disease-specific immunosuppression. Notably, the colony stimulating factor 1 receptor (CSF1R), which is highly expressed on pre-transplant M-MDSCs and binds to its ligand CSF1, has been linked to impaired melphalan cytotoxicity and predicts inferior clinical outcomes following stem cell transplantation in MM patients (28).

#### 3.2.2 Regulation by the tumor microenvironment

The fate of MDSCs recruited into the TME is further shaped by local conditions (79), among which hypoxia is a key driver that enhances MDSCs recruitment and fosters an immunosuppressive, pro-angiogenic niche (80, 81). Hypoxia-inducible factor-1α (HIF-1α) plays a central role in this process (82). In MM, emerging evidence demonstrates that hypoxia induces HIF-1α to drive MM-associated bone disease and contributes to drug resistance and metastasis (83–85). These observations suggest that the hypoxic bone marrow niche may similarly promote MDSCs accumulation and function in MM, although direct mechanistic evidence remains limited.

#### 3.2.3 Subset-specific differentiation pathways

Emerging evidence highlights functional divergence between MDSCs subsets. A recent study demonstrated that macrophages derived from M-MDSCs exhibit potent immunosuppressive activity and constitutively express S100A9, a feature absent in tissue-resident and monocyte-derived macrophages (86). Notably, due to their shorter lifespan compared to M-MDSCs, the differentiation mechanisms of PMN-MDSCs within the TME remain poorly understood (87). In MM, G-CSF administration significantly increases myeloid-derived suppressor cells (MDSCs) subsets, inducing a transient rise in circulating M-MDSCs and PMN-MDSCs (27, 88). This effect is further supported by studies showing that short-term G-CSF exposure is associated with elevated M-MDSCs levels in MM patients (89). These findings highlight the dynamic plasticity of MDSCs subsets in response to therapeutic interventions and the TME, directly linking their biology to clinical disease management.

Overall, MDSCs differentiation in the tumor niche is complex, and the specific drivers of M-MDSCs versus PMN-MDSCs divergence remain unclear. Further research using multi-omics and single-cell sequencing is needed to elucidate these mechanisms and inform MDSC-targeted immunotherapy.

## 4 Interaction between MDSCs and myeloma

### 4.1 Mechanisms of MDSC-mediated immunosuppression

MDSCs exert immunosuppressive and tumor-promoting functions within the TME through a variety of mechanisms (10, 90), which primarily include: 1) Regulation of amino acid metabolism: By activating enzymes such as arginase-1 (ARG-1), inducible nitric oxide synthase (iNOS), and indoleamine 2,3-dioxygenase 1 (IDO), MDSCs deplete essential amino acids including L-arginine and L-tryptophan, thereby inhibiting T-cell proliferation and activation (91, 92). 2) Release of immunosuppressive cytokines: MDSCs secrete cytokines such as interleukin-10 (IL-10) and transforming growth factor-β (TGF-β), which promote the differentiation and expansion of regulatory B cells (Bregs) and regulatory T cells (Tregs) (93–95). Additionally, these cytokines facilitate the recruitment of Tregs to the tumor site (96). 3) Regulation of immune checkpoints: MDSCs upregulate programmed death ligand-1 (PD-L1) expression, which engages programmed death 1 (PD-1) on T cells and suppresses antitumor T-cell responses (97, 98). This process is associated with elevated levels of HIF-1α (99). M-MDSCs primarily function through surface inhibitory molecules such as PD-L1 (13, 100, 101), whereas PMN-MDSCs tend to induce T-cell apoptosis via the Fas/FasL pathway (102). 4) Interference with immune cell function: MDSCs downregulate activating receptors on NK cells and impair the antigen-presenting capacity of dendritic cells (103, 104). Furthermore, they induce M2 macrophage polarization and promote further MDSCs recruitment through mechanisms that include downregulation of STAT-3, elevation of HIF-1α, and secretion of S100A8/9 (105, 106).

Due to subset-specific differences, MDSCs counteract antitumor immune responses through distinct mechanisms (13). For instance, M-MDSCs primarily mediate immunosuppression by expressing high levels of TGF-β, IL-10, iNOS, and PD-L1, whereas PMN-MDSCs exert their inhibitory effects largely through the production of substantial amounts of reactive oxygen species (ROS), ARG-1, and prostaglandin E2 (PGE2) (**Table 2**) (17), which summarizes the key mechanistic pathways. The relative contribution and redundancy among these immunosuppressive pathways remain unclear, and most findings derive from in vitro or murine systems. Patient-level functional prioritization of these axes is urgently needed.

**Table 2:** Mechanisms of bidirectional interaction between MDSCs and the MM tumor microenvironment.

MM cells → MDSCs (Induction of recruitment, survival, and activation)			
Acting cell/key factor	Target cell/signaling pathway	Functional outcome	Remarks
MM cells secrete IL-6	MDSCs, STAT3 pathway and induce expression of the anti-apoptotic protein Mcl-1 (apoptotic pathway)	• Blocks MDSCs differentiation, promoting their accumulation (111, 112).	*In vitro* evidence only; redundancy with GM-CSF unclear.
MIF, highly expressed in MM patients	MDSCs (CD84 signaling pathway → PD-L1 expression)	• Upregulates PD-L1 expression on MDSCs, enhancing their immunosuppressive activity (114, 115).	Correlative patient data; lacks *in vivo* functional validation.
MM cells secrete CCL5, MIF	Myeloid precursor cells	• Serves as a prerequisite for MDSCs generation, promoting their production (58).	Relative contribution of each chemokine unknown.
MM cells secrete Galectin-1	MDSCs (via CD304 receptor)	• Enhances the immunosuppressive function of MDSCs (116, 117).	CD304 blockade untested in MM models.
MM cells release exosomes (containing miR-106a-5p, miR-146a-5p)	Myeloid precursor cells	• Cooperates with CCL5 and MIF to induce M-MDSCs generation (118).	Packaging mechanisms and subtype specificity unknown.
MM cells release exosomes	upregulate iNOS	• Enhances the immunosuppressive function of MDSCs (119).	iNOS upstream signaling in MDSCs uncharacterized.
Bone marrow stromal cells secrete IL-6, IL-10, TGF-β, PTGS2, etc.	MDSCs	• Synergistically induces and activates immunosuppressive MDSCs (29, 124).	Hierarchy of BMSC vs. MM-derived signals unclear.
IL-18 derived from the MM microenvironment (mouse model)	MDSCs	• Promotes MDSCs generation and expansion, accelerating disease progression (125).	Murine data only; human relevance unconfirmed.

MDSCs → MM cells and microenvironment (Promoting growth, drug resistance, and immune evasion)			
Acting cell/key factor	Target cell/signaling pathway	Functional outcome	Remarks
PMN-MDSCs trigger piRNA-823 expression	MM cells (epigenetic regulation)	• Promotes DNA methylation, enhancing the stemness and tumorigenic potential of MM cells (31).	Upstream regulators of piRNA-823 in MDSCs unknown.
MDSCs promote via AMPK phosphorylation	MM cells (metabolism)	• Enhances β-oxidation, ATP production in MM cells, sustaining their survival (128).	*In vivo* contribution to drug resistance unconfirmed.
MDSCs secrete S100A9	MM cells (via TLR4/NF-κB pathway)	• Induces pro-inflammatory factor release in an autocrine manner, recruiting MM cells and promoting their proliferation (129, 130).	RAGE vs. TLR4 contribution in MM unclear.
PMN-MDSCs	Tumor microenvironment	• Promotes angiogenesis (131).	Angiogenic factors in MM not characterized.
MDSCs	T cells	• Facilitates the differentiation of regulatory T cells (Tregs) and suppresses T-cell proliferation (58, 113).	Contact-dependent vs. soluble-mediated debate unresolved.
MDSCs	Osteoclasts	• Transdifferentiate into functional osteoclasts, promoting bone sdisease (135).	*In vivo* contribution to bone disease unquantified.

MM, multiple myeloma; MDSCs, myeloid-derived suppressor cells; M-MDSCs, monocytic MDSCs; PMN-MDSCs, polymorphonuclear MDSCs; IL, interleukin; STAT3, signal transducer and activator of transcription 3; Mcl-1, induced myeloid leukemia cell differentiation protein 1; MIF, macrophage migration inhibitory factor; PD-L1, programmed death-ligand 1; Tregs, regulatory T cells.

Beyond PD-1/PD-L1, other checkpoint molecules are aberrantly expressed in the MM microenvironment. VISTA, a PD-L1 homolog predominantly expressed on myeloid cells and Tregs, shows elevated co-expression with PD-1, TIM-3, and TIGIT on T cells in MM patients, correlating with T-cell exhaustion and disease progression (107). LAG-3 and TIM-3 are also significantly upregulated on T cells in active MM, and their blockade has been shown to enhance myeloma-specific cytotoxic T-cell function (107, 108). Moreover, an immunometabolic checkpoint links MDSC-mediated arginine deprivation to checkpoint regulation: MDSC-derived ARG-1 depletes arginine, triggering the GCN2 integrated stress response that sustains PD-1 expression on MM cells—a mechanism reversible upon ARG-1 inhibition (109, 110).

### 4.2 Myeloma-mediated regulation of MDSCs

Given their potent immunosuppressive capabilities, the accumulation and sustained activation of MDSCs within the MM microenvironment are not passive events but are actively driven by MM cells themselves. MM cells can induce the development of MDSCs, which in turn promote tumor growth, confer resistance to chemotherapy, and facilitate immune evasion (**Table 2**) (10). The development and immunosuppressive functions of MDSCs are supported through both direct and indirect mechanisms mediated by MM cells and neighboring cells in MM TME. Direct mechanisms orchestrated by MM cells include the following: 1) MM cell-derived IL-6 activates JAK2/STAT3 signaling and upregulates anti-apoptotic proteins including Mcl-1 (111). IL-6 is a key regulator of MDSCs accumulation in the TME (112), and STAT3 signaling is critically involved in MDSCs expansion and function (38). MM cells also upregulate Mcl-1 to promote MDSCs survival (113). Together, these mechanisms promote MDSCs accumulation. 2) MIF is highly expressed in MM patients and correlates positively with MDSCs levels in the BM microenvironment (114). It enhances the immunosuppressive activity of MDSCs by upregulating PD-L1 expression via the CD84 pathway (115). 3) Expression of CCL5 and macrophage migration inhibitory factor (MIF), which are prerequisites for MM-induced MDSCs generation (58). 4) Secretion of galectin-1, which enhances the immunosuppressive function of MDSCs by targeting the CD304 receptor on their surface (116, 117). 5) Release of exosomes: Emerging evidence indicates that exosomes derived from different cellular sources exert distinct effects on MDSCs in the MM microenvironment. MM cell-derived exosomes, containing miR-106a-5p and miR-146a-5p, cooperate with CCL5 and MIF to promote the generation of M-MDSCs (118), and can also upregulate iNOS to enhance the immunosuppressive function of MDSCs MDSCs in vivo (119). In addition, MM cell-derived exosomes promoted IL-6 secretion and suppressed osteoblastic differentiation and mineralization of BMSC (120, 121). In contrast, BMSC-derived exosomes promote MDSCs survival via STAT3/STAT1 pathway activation and upregulation of the anti-apoptotic proteins Bcl-xL and Mcl-1 (122). These distinct mechanistic pathways underscore the need to consider exosomal origin when evaluating MDSC-targeting strategies in MM. 6) In ultra-high-risk MM patients, malignant plasma cells exhibit elevated expression of the immune checkpoint molecule LILRB4. Genetic deletion of LILRB4 not only inhibits MM cell growth but also directly attenuates the immunosuppressive function of MDSCs, uncovering a critical cell-surface-mediated pathway for TME modulation (123).

In addition to the mechanisms by which MM cells induce MDSCs, recent research has expanded to examine the direct or indirect influence of stromal cells on MDSCs. Neighboring cells within the TME also cooperate with MM cells to induce and activate immunosuppressive MDSCs. These mechanisms include: 1) Production and release of immunosuppressive molecules by BMSC and osteoblasts, such as prostaglandin-endoperoxide synthase 2 (PTGS2), TGF-β, nitric oxide synthase 2 (NOS2), IL-10, and IL-6 (29, 124). 2) Interleukin-18 (IL-18) derived from the MM microenvironment promotes the generation and expansion of MDSCs, thereby accelerating disease progression (125).

### 4.3 MDSC-mediated mechanisms of myeloma progression

The accumulation and activation of MDSCs create a potent pro-tumorigenic loop, conferring a survival advantage to myeloma cells (126, 127). The specific mechanistic contributions of MDSCs include the following: 1) Epigenetic modulation enhances myeloma stemness: PMN-MDSCs trigger the expression of piRNA-823, which promotes DNA methylation and increases the tumorigenic potential of MM cells (31). 2) Metabolic reprogramming supporting MM survival: MDSCs enhance β-oxidation, ATP production, and NADPH levels in MM cells via AMPK phosphorylation, thereby sustaining their viability (128). 3) Secretion of pro-tumorigenic mediators: MDSC-derived S100A9 acts in an autocrine manner through the TLR4 pathway to induce the release of TNF-α, IL-6, and IL-10. This recruits MM cells to the TME and promotes their proliferation by activating the canonical NF-κB pathway (129, 130).

Furthermore, MDSCs indirectly support MM progression through several mechanisms: PMN-MDSCs promote angiogenesis (131); MDSCs facilitate the differentiation of Tregs (58) and suppress T-cell proliferation (113); specifically, M-MDSCs and CD84+ MDSCs have been shown to significantly inhibit the activation and cytotoxic cytokine production of CD8+ T cells in vitro, directly impairing the primary adaptive anti-tumor response (132); MDSCs enhance the immunosuppressive activity of Bregs against T cells via the PD-1/PD-L1 axis (133, 134); MDSCs can transdifferentiate into functional osteoclasts (135); MDSCs also actively contribute to establishing the immunosuppressive MM microenvironment (136). A central theme across these mechanisms is the suppression of anti-myeloma immune responses, establishing a bidirectional pro-tumorigenic network between MDSCs and MM cells.

## 5 Therapeutic strategies targeting MDSCs

Immunotherapy is recognized as an effective approach for treating MM. The mechanisms of interaction between MM and MDSCs outlined above provide a strong theoretical rationale for developing MDSC-directed immunotherapeutic strategies. However, the clinical translation of MDSC-targeting approaches in MM is still in its early stages. Current research encompasses both MM-specific investigations and insights borrowed from other malignancies, offering valuable mechanistic paradigms for exploration in MM. Several novel agents are in preclinical and clinical development, and certain conventional drugs have also been shown to modulate MDSCs levels. Therapeutic strategies targeting MDSCs can be broadly categorized into four complementary approaches: depleting MDSCs, inducing their differentiation, blocking their recruitment, and inhibiting their immunosuppressive function (summarized in **Table 3**; **Figure 2**) (7, 11). The overarching goal of these strategies is to alleviate the immunosuppressive TME and thereby synergistically enhance the efficacy of established and emerging anti-MM immunotherapies (9, 137).

**Table 3:** Overview of major therapeutic strategies targeting or influencing MDSCs.

Strategy category	Target/mechanism of action	Representative drugs/interventions	Key findings (related to MM/cancer)	Remarks
1. Depleting MDSCs	Cell Cycle/Apoptosis (Non-specific)	5-Fluorouracil, Gemcitabine, Paclitaxel	• Depletes MDSCs in the OB- Runx2-/- mouse MM model, reversing immunosuppression and bortezomib resistance, and suppressing tumor growth (138); • demonstrates efficacy and tolerability in RRMM patients (139, 140); • Paclitaxel delivered via nanoparticle- based systems decreases MDSCs proportion and demonstrates anti- myeloma activity (141, 142).	Non-selective; narrow therapeutic window; no MM-specific biomarkers.
	CD33	Gemtuzumab ozogamicin	• Effectively depletes CD33-expressing MDSCs in phase II clinical trials for acute leukemia, correlating with therapeutic efficacy (143).	CD33 also on normal progenitors; no MM clinical data.
	TRAIL-R2	DS-8273a	• Induces selective reduction of MDSCs in a phase I trial of patients with advanced cancers, associated with improved progression-free survival (144).	Solid tumor data only; TRAIL-R2 on MM-MDSCs uncharacterized.
	Liver X Receptor (LXR)	GW3965, RGX-104	• Induces MDSCs apoptosis, depletes MDSCs, and inhibits tumor growth in preclinical models (145).	MM-specific preclinical data absent; pleiotropic metabolic effects.
2. Inducing MDSCs differentiation	Retinoic Acid Pathway	All-Trans Retinoic Acid (ATRA)	• Promotes MDSCs differentiation in preclinical models; enhances anti- angiogenic efficacy of VEGFR2 inhibitors by inducing MDSCs differentiation (146, 147); • In MM research, it upregulates BCMA expression on MM cells and enhances the responsiveness of BCMA-CAR T cells (148).	Preclinical tumor models; MM cells *in vitro*
	Vitamin D Receptor	Vitamin D3	• Triggers MDSCs differentiation; induces epigenetic modifications in MM cells *in vitro* and may help prevent bortezomib resistance; vitamin D deficiency in MM patients is associated with poorer prognosis, suggesting its potential therapeutic value (149–151).	*In vitro* MM cells; MM patients (observational)
	Bisphosphonate Action	Zoledronic Acid	• Reduces intratumoral MDSCs accumulation and enhances anti- tumor immunity in a murine pancreatic cancer model. In MM, it is primarily used to treat bone disease, but may exert a dual effect by indirectly potentiating anti- tumor immunity (152, 153).	MDSCs effect not studied in MM; contribution to efficacy unclear.
3. Disrupting MDSCs recruitment	CXCR2 Chemokine Receptor	CXCR2 Inhibitors	• Blocks PMN-MDSCs migration in a murine sarcoma model, improves prognosis, and enhances the efficacy of anti-PD-1 therapy (154, 155).	Non-MM model; CXCR2 also on neutrophils—safety concerns.
	S100A9/RAGE/TLR4 Pathway	Tasquinimod	• Preclinical studies show it suppresses MM cell proliferation, impairs MDSCs function, and prolongs survival in MM-bearing mice (157, 158); • Currently undergoing an Ib/IIa phase combination therapy trial in RRMM patients (NCT04405167); definitive clinical outcomes pending (158, 159).	MM mouse models; RRMM patients (Phase 1b/IIa)
4. Inhibiting MDSCs function	PI3K/AKT/mTOR Pathway	Duvelisib, Idelalisib	• Suppresses MDSCs function and delays tumor growth in a murine head and neck cancer model (161); • Currently in phase II trials for lymphocytic leukemia (NCT04209621) and Hodgkin’s lymphoma (NCT01393106), respectively.	Head and neck cancer model(no MM data)
	JAK/STAT Pathway (STAT3)	AZD9150, Napabucasin	• AZD9150 reduces PMN-MDSCs and increases T cells in a small cohort of NHL patients (164); • Napabucasin abolishes MDSCs function and improves survival in preclinical models (165).	Small cohorts/non-MM models; off-target concerns.
	Arginase-1 (ARG-1)	nor-NOHA, INCB001158	• Restores T-cell responsiveness in MM patients (166); • Restores T-cell proliferation and delays tumor progression in MM mouse models (167).	MM patients *in vitro*; MM mouse models.
	Phosphodiesterase-5 (PDE5)	Sildenafil, Tadalafil	• Downregulates ARG-1 and iNOS in MDSCs in preclinical models, impairing their function (168); • A case report showed tadalafil combination reduced MDSCs activity and achieved deep response (169); • A combination trial in lenalidomide- refractory patients was terminated due to lack of efficacy (170).	Preclinical tumor models; MM patient case report; MM patients (Phase II, negative)
	Cyclooxygenase-2 (COX2)	COX2 Inhibitors	• Blocks MDSCs expansion in preclinical models (non-MM) (171); • In MM, targeting COX2 in BMSCs may inhibit tumor cell proliferation and adhesion (172).	Broad systemic effects; MDSCs specificity not achieved.
	Indoleamine 2,3-Dioxygenase (IDO)	IDO Peptide Vaccine	• Reduces IDO^+^ MDSCs and Tregs in tumor-bearing mice, improving therapeutic efficacy (173).	Early preclinical; no MM data.
	Histone Deacetylase (HDAC)	Decitabine, ACY241	• In a mouse MM model, depletes M- MDSCs and increases T-cell infiltration, suppressing tumor proliferation (175); • ACY241 reduces the proportion of MDSCs in MM patient samples *in vitro* (176).	MM mouse models; MM patient BM samples *in vitro*; MDSCs effect likely indirect;
5. Impact of standard therapies on MDSCs	Proteasome	Bortezomib	• Reduces M-MDSCs levels in co- culture assays with MM cells *in vitro* (24); • Combination with an S100A9 inhibitor enhances tumor burden reduction (MM mouse model) (129).	*In vivo* MDSCs effect in patients not well documented.
	Immunomodulation	Lenalidomide, Pomalidomide	• Reduces circulating ARG-1 and PMN-MDSCs levels in MM patients (32); • Combination with immunotherapy in preclinical MM models reduces MDSCs and enhances anti-tumor immunity in MM patients (58, 178 180).	MDSCs effect may be indirect via T-cell/NK-cell activation.
	CD38 antibody	Daratumumab	• Exerts ADCC/CDC-mediated killing against CD38-high PMN-MDSCs *in vitro* MM models (177); clinical combination regimens selectively reduce M-MDSCs in MM patients (182).	CD38 also on activated T/NK cells; net effect may vary.

MDSCs, myeloid-derived suppressor cells; MM, Multiple myeloma; RRMM, relapsed/refractory multiple myeloma; BCMA, B-cell maturation antigen; CAR-T, chimeric antigen receptor T-cell; PMN-MDSCs, polymorphonuclear MDSCs; M-MDSCs, monocytic MDSCs; PD-1, programmed cell death protein 1; NHL, non-Hodgkin lymphoma; BMSC, bone marrow stromal cell; Treg, regulatory T cell; HDAC, histone deacetylase; ADCC, antibody-dependent cellular cytotoxicity; CDC, complement-dependent cytotoxicity.

**Figure 2:**
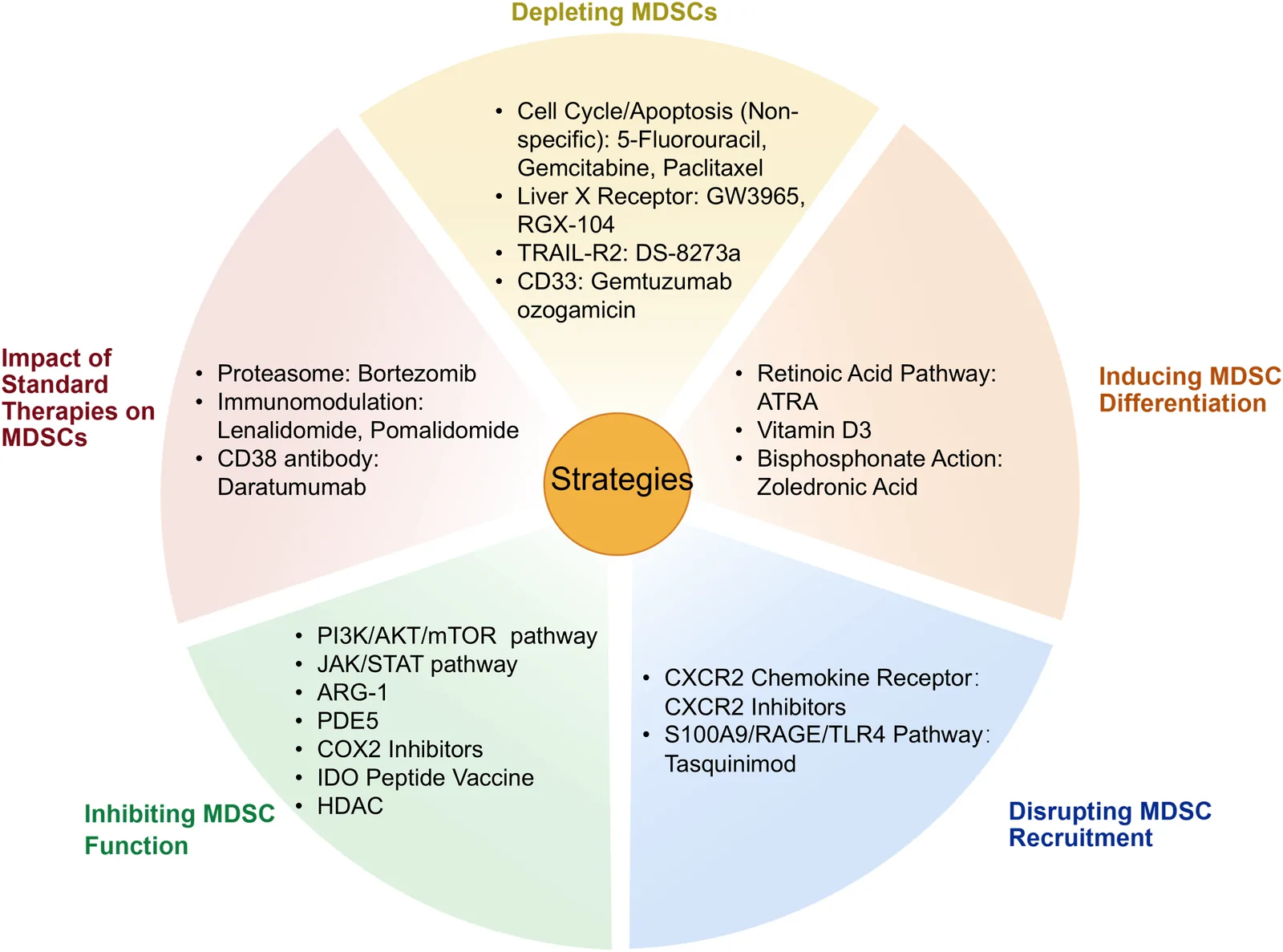
Therapeutic strategies targeting or influencing MDSCs. Current clinical strategies targeting MDSCs in MM primarily focus on four aspects: inducing MDSCs differentiation, disrupting MDSCs recruitment, inhibiting MDSCs immunosuppressive function, and modulating MDSCs through standard anti-myeloma therapies. Created with BioGDP.com.

### 5.1 Strategies to deplete MDSCs

MDSCs are short-lived cells that require constant renewal and release into the circulation. Although depleting these highly proliferative cells presents challenges, multiple studies have demonstrated that conventional chemotherapeutic agents such as 5-fluorouracil, gemcitabine and paclitaxel can effectively eliminate MDSCs by interfering with the cell cycle or inducing apoptosis. In a murine MM model with osteoblast (OB)-specific deletion of Runt-related transcription factor 2 (Runx2-/-), treatment with 5-fluorouracil or gemcitabine reversed the OB-Runx2 deficiency-induced immunosuppressive microenvironment and bortezomib resistance by depleting MDSCs, leading to significant tumor suppression (138). Clinical studies have shown that gemcitabine-based regimens exhibit certain efficacy and favorable tolerability in patients with RRMM (139, 140). Paclitaxel, especially when delivered via nanoparticle systems (e.g., Rg3-lipo or other targeted carriers), can consistently decrease circulating MDSCs and exhibits anti-myeloma activity (141, 142).

However, these chemotherapeutic agents lack specificity and also affect rapidly dividing anti-tumor T cells. Therefore, more targeted biological approaches are being developed. For example: i) CD33-targeted therapy: The anti-CD33 antibody-drug conjugate gemtuzumab ozogamicin effectively depletes CD33-expressing MDSCs, correlating with therapeutic efficacy in acute leukemia trials (143) (evidence from other hematologic malignancies; MM validation is lacking). ii) TRAIL-R2 agonism: The TRAIL-R2 agonist antibody DS-8273a induced selective MDSCs reduction in patients with advanced cancers and was associated with improved progression-free survival (144), but not yet in MM. iii) Liver X receptor (LXR) activation: LXR agonists (e.g., GW3965, RGX-104) trigger MDSCs apoptosis via ApoE induction and show potent MDSC-depleting and tumor-inhibitory effects in non-MM preclinical models (145).

### 5.2 Inducing differentiation of MDSCs

Vitamin A and its derivatives can promote the differentiation of MDSCs (146). In preclinical tumor models, all-trans retinoic acid (ATRA) enhances the antiangiogenic efficacy of vascular endothelial growth factor receptor 2 (VEGFR2) inhibitors by inducing MDSCs differentiation, leading to a significant delay in tumor growth (147). In MM research, ATRA upregulates the expression of B-cell maturation antigen (BCMA) on MM cells, thereby enhancing the responsiveness of BCMA-targeted chimeric antigen receptor T (CAR T) cells (148).

Vitamin D3 can also trigger MDSCs differentiation (149). In vitro studies indicate that vitamin D induces epigenetic modifications in MM cells and may partially prevent the development of bortezomib resistance (150). Clinical data reveal that MM patients with vitamin D deficiency have a poorer prognosis, suggesting its potential therapeutic value (151). By modulating both MDSCs differentiation and the epigenetic state of tumor cells, vitamin D offers a novel strategy to delay drug resistance and improve outcomes.

Zoledronic acid, a bisphosphonate, is currently used primarily to manage MM-related bone disease (152). Although its potential for targeting MDSCs in hematologic malignancies remains unclear, studies in a murine pancreatic cancer model have shown that zoledronic acid reduces intratumoral MDSCs accumulation and enhances antitumor immune responses (153). This implies that zoledronic acid may exert a dual effect in MM therapy—ameliorating bone disease while indirectly potentiating antitumor immunity.

### 5.3 Disrupting MDSCs recruitment

CXCR2 plays a pivotal role in the recruitment of MDSCs to tumor sites (154). Early studies demonstrated that blocking the migration of PMN-MDSCs—either through CXCR2 gene knockout or via small-molecule CXCR2 inhibitors—significantly improved outcomes in metastatic sarcoma models and enhanced the efficacy of anti-PD-1 therapy against established tumors (155) (solid tumor evidence; relevance to MM remains to be confirmed).

S100A9, a calcium-binding protein, promotes MDSCs recruitment into the TME by interacting with its receptors RAGE and TLR4 (156). Tasquinimod, a small-molecule inhibitor targeting this pathway, is currently being evaluated in an Ib/IIa phase clinical trial for MM (NCT04405167). Preclinical studies have shown that tasquinimod effectively suppresses MM cell proliferation and clonogenicity in vitro, impairs the immunosuppressive function of MDSCs, and promotes T-cell proliferation. In vivo, it extended OS in MM mouse models (157). Tasquinimod was evaluated in a Phase Ib/IIa trial (NCT04405167) combined with ixazomib, lenalidomide, and dexamethasone (IRd) in RRMM patients (158). At ASCO 2025, the final analysis of 17 heavily pretreated patients (median 7 prior lines, all triple-class refractory) reported a 47% clinical benefit rate, with one durable partial response (19.8 months) and three minimal responses in patients refractory to their most recent IMiD/PI-based regimen. The combination was well tolerated with no dose-limiting toxicities, and the study has closed to enrollment (159) (**Table 4**).

**Table 4:** Clinical trials of MDSC-modulating strategies in MM.

Drug (target)	Combination	Phase/population	Key findings/status
Tasquinimod (S100A9 inhibitor)	+ Ixazomib + Lenalidomide + Dexamethasone (IRd)	Phase 1b/IIa (NCT04405167)/RRMM, median 7 prior lines, triple-class refractory	47% clinical benefit rate (1 PR + 3 MR) (158, 159); 1 durable PR lasting 19.8 months; Phase IIa enrolling
Tadalafil (PDE5 inhibitor)	+ Lenalidomide + Dexamethasone	Phase II/Lenalidomide-refractory MM	Case report: ~90% tumor reduction (169); subsequent trial terminated early due to lack of efficacy, underscoring need for biomarker-driven selection (170)
Pembrolizumab (Anti-PD-1)	+ Lenalidomide + Dexamethasone (KEYNOTE-185)	Phase III/Newly diagnosed, transplant-ineligible MM	Enrollment suspended due to higher mortality in pembrolizumab arm (6.7% vs 0.6%) (190); monotherapy showed some efficacy in consolidation (191, 192)
Isatuximab (Anti-CD38)	+ ASCT	Phase II (NCT05346809)/MM undergoing ASCT	Trend toward improved Day 30 ALC; favorable immune repertoire changes, suggesting potential MDSCs modulation (183)

PR, partial response; MR, minimal response; RRMM, relapsed/refractory multiple myeloma; IRd, ixazomib + lenalidomide + dexamethasone; ASCT, autologous stem cell transplantation; ALC, absolute lymphocyte count; PDE5, phosphodiesterase type 5; PD-1, programmed cell death protein 1.

Thus, blocking key recruitment pathways such as CXCR2 and S100A9/RAGE/TLR4 offers a promising strategy to reduce MDSCs infiltration and potentially enhance immunotherapy efficacy in MM.

### 5.4 Inhibition of MDSCs immunosuppressive activity

Multiple pharmacologic strategies aim to directly inhibit the immunosuppressive machinery of MDSCs, targeting key signaling nodes and metabolic enzymes within the TME.

#### 5.4.1 Targeting key oncogenic pathways

In MM, the PI3K/AKT/mTOR signaling pathway is frequently hyperactivated due to oncogenic events and exogenous stimuli, contributing to tumor progression and therapy resistance (160). Recent studies in a murine head and neck cancer model have shown that PI3K inhibitors can suppress MDSCs function and delay tumor growth (161), a finding that awaits confirmation in MM. While not yet evaluated in MM, the PI3K inhibitor duvelisib (in combination with ibrutinib) is undergoing a phase II trial for lymphocytic leukemia (NCT04209621), and idelalisib is in a phase II trial for Hodgkin lymphoma (NCT01393106).

Abnormal activation of the JAK/STAT pathway has been identified as a key driver in MM progression (162). STAT3 activation plays a particularly critical role in mediating the recruitment, activation, and functional maintenance of MDSCs (163). In a small cohort of patients with non-Hodgkin lymphoma, the STAT3 mRNA-targeting inhibitor AZD9150 reduced peripheral PMN-MDSCs and increased CD4+ and CD8+ T-cell counts in three out of four patients (164). Another STAT3 inhibitor, napabucasin, completely abrogated the immunosuppressive function of both mouse and human MDSCs and significantly improved survival in melanoma-bearing mice (165); its effects in MM models have not been reported.

#### 5.4.2 Inhibiting immunosuppressive enzymes

ARG-1, highly expressed by PMN-MDSCs, depletes arginine in the microenvironment and suppresses T-cell function. Pharmacologic inhibition of ARG-1 with agents such as nor-NOHA or INCB001158 can significantly restore T-cell responsiveness in MM patients (166). Research in MM-bearing mice demonstrated that genetic deletion or pharmacological inhibition of ARG-1 restored T-cell proliferation and significantly delayed tumor progression in the Vk*MYC model (167).

Phosphodiesterase-5 (PDE5) inhibitors (e.g., sildenafil, tadalafil), though clinically used for non-malignant conditions, exhibit immunomodulatory effects in preclinical tumor models. For instance, sildenafil downregulates ARG-1 and iNOS expression in MDSCs, thereby attenuating their immunosuppressive function and enhancing antitumor immunity (168). Although their direct application in MM has not been extensively studied, their capacity to remodel immune responses within the TME warrants further investigation. A case report of a patient with advanced RRMM documented that the addition of tadalafil to the treatment regimen (lenalidomide, clarithromycin, dexamethasone) reduced MDSCs inhibitory activity, as evidenced by decreased levels of IL4Rα^+^, iNOS, ARG-1, and ROS, alongside enhanced T-cell function (increased IFN-γ expression). The patient tolerated the combination well and achieved a very good partial response, with a reduction in tumor burden of approximately 90% (169). However, a subsequent clinical trial combining tadalafil, dexamethasone, and lenalidomide in lenalidomide-refractory MM patients was terminated early due to a lack of efficacy (170) (**Table 4**). This outcome suggests that biomarker-guided patient selection—rather than unselected enrollment—is critical for future MDSC-targeted trials. This may be explained by the immunomodulatory effect of prior lenalidomide treatment, which can lower baseline MDSCs levels and thereby diminish the potential benefit of subsequent MDSC-targeted therapy (170). Thus, PDE-5 inhibitors may be most effective in patients with elevated MDSCs levels, highlighting the need for biomarker-guided patient selection in future studies.

#### 5.4.3 Modulating inflammatory and epigenetic axes

Systemic inhibition of PGE2 via cyclooxygenase-2 (COX2) inhibitors has been shown to suppress MDSCs expansion in non-MM murine tumor models (171); however, whether this strategy translates to MM remains to be determined. In MM, COX2 promotes the proliferation and adhesion of MM cells induced by BMSCs to increase the secretion of PGE2 and TNF-α (172). Consequently, targeting COX2 in BMSCs represents a potential therapeutic strategy for MM.

IDO, a potent immunosuppressive enzyme primarily produced by tumor cells and MDSCs, is a key component of the TME. In two different non-MM murine tumor models, treatment with an IDO-specific peptide vaccine (IDO vaccine) significantly reduced the numbers of both IDO^+^ MDSCs and regulatory T cells (Tregs), thereby enhancing therapeutic efficacy (173) (preclinical evidence from solid tumors; no MM data available).

Histone deacetylase inhibitors (HDACi) have become an integral component of combination regimens in MM therapy (174). In a mouse MM model, treatment with decitabine suppressed the in vivo proliferation of MPC-11 MM tumor cells by depleting M-MDSCs and increasing T-cell infiltration into the tumor tissue (175). Furthermore, In vitro studies further show that the HDAC inhibitor ACY241 reduces not only CD138^+^ MM cells but also the frequencies of Tregs and MDSCs in MM patient BM samples (176).

In summary, inhibiting MDSCs function through diverse mechanisms—spanning signal transduction, metabolism, inflammation, and epigenetics—represents a multifaceted approach to reverse immunosuppression in MM. The clinical translation of these strategies will require careful patient selection and rational combination with therapies that modulate the MDSCs compartment.

### 5.5 Combining MDSC-targeted therapies with standard myeloma regimens

Standard therapeutic agents for MM, including bortezomib, immunomodulatory drugs (IMiDs; lenalidomide and pomalidomide), and anti-CD38 monoclonal antibodies, have been shown to modulate the abundance and function of MDSCs (177).

#### 5.5.1 Proteasome inhibitors

Wang et al. demonstrated that coculture of MM cells with PB mononuclear cells in the presence of bortezomib significantly reduced M-MDSCs levels compared to untreated controls (24). Furthermore, combining bortezomib with the S100A9 inhibitor ABR-238901 resulted in greater tumor burden reduction than bortezomib alone (129). There are currently no reported studies on the relationship between carfilzomib and MDSCs.

#### 5.5.2 Immunomodulatory drugs

Treatment with lenalidomide in MM patients significantly reduces circulating levels of ARG-1 and PMN-MDSCs (32). In a murine MM model, combining lenalidomide with DC vaccination and anti-PD-1 therapy reduced MDSCs numbers, enhanced cytotoxic T lymphocyte and NK cell activity, and demonstrated superior anti-myeloma efficacy (178). Pomalidomide a third-generation IMiD, has been widely utilized in treating MM (179). Pomalidomide can also reduce MDSCs (58, 180).

#### 5.5.3 CD38 antibody

Daratumumab, an anti-CD38 monoclonal antibody, approved by the U.S. Food and Drug Administration in 2015 for the treatment of RRMM, targets not only MM cells but also MDSCs and Bregs, which express CD38 (181). In vitro studies have shown that PMN-MDSCs derived from cocultures of healthy donor PB mononuclear cells and MM cell lines express high levels of CD38 and are highly susceptible to daratumumab-mediated antibody-dependent cellular cytotoxicity and complement-dependent cytotoxicity (181). A corresponding effect was observed clinically: in patients treated with the combination of lenalidomide, dexamethasone, and daratumumab, a selective reduction in M-MDSCs was noted compared to those receiving only lenalidomide and dexamethasone (182). Similarly, isatuximab, another anti-CD38 antibody, has shown preliminary immunomodulatory effects in a Phase II trial (NCT05346809) when combined with ASCT, with trends toward improved Day 30 lymphocyte recovery and favorable immune repertoire changes, suggesting potential MDSCs modulation (183) (**Table 4**). T-cell engaging bispecific antibodies targeting the CD38 antigen may help delay disease progression in MM by depleting both the malignant MM clones and the MDSCs in the BM microenvironment (184).

### 5.6 Synergy with immunotherapy

Advances in targeted therapy underscore that effective cancer treatment strategies must not only act directly on tumor cells or stromal components but also dismantle the blockade against antitumor immune responses. MDSCs, as a population of aberrantly accumulated myeloid immune cells in the TME, suppress immune function and promote tumor progression through diverse mechanisms. Consequently, disrupting MDSC-mediated immunosuppression has emerged as a pivotal strategy for remodeling the immune microenvironment and enhancing antitumor efficacy (185, 186).

#### 5.6.1 PD-1/PD-L1 inhibitors

In MM, PD-L1 expression is significantly elevated on PMN-MDSCs from the BM and PB of newly diagnosed and relapsed patients compared to those from individuals with MGUS (30). Studies indicate that combining lenalidomide with PD-1/PD-L1 blockade can inhibit MDSC-mediated immunosuppression (187). In the MOPC-315 MM model, a novel anti-PD-L1 antibody containing a mouse IgG2a Fc domain demonstrated significant antitumor activity, which may be associated with a reduction in MDSCs; however, no significant change in splenic MDSCs numbers was observed upon its combination with lenalidomide (188). In a study on RRMM by Cohen et al., daratumumab was initially combined with the anti-PD-1 antibody cetrelimab (189). Following early termination of cetrelimab in external trials, patients continued on daratumumab monotherapy, which resulted in a significant decreasing trend in PB CD38^+^ M-MDSCs (189). In another MM clinical trial, patient enrollment for studies involving the PD-1 inhibitor pembrolizumab was suspended due to immune-related toxicities and mortality concerns (190). Subsequent studies, however, indicated that pembrolizumab monotherapy still exhibited certain efficacy and was tolerable (191, 192) (**Table 4** and **Table 5**). Collectively, these observations underscore a key mechanistic insight: depletion of MDSCs may enhance the sensitivity of patients with MM to PD-1 immune checkpoint blockade therapy (132).

**Table 5:** Synergistic effects of MDSC-targeting strategies with immunotherapy.

Category/strategy	Mechanism of action/target	Key preclinical/clinical findings	Evidence source	Implications for MM therapy
Synergy with PD-1/PD-L1 inhibitor	Targeting PD-L1 MDSCs in MM microenvironment	• PMN-MDSCs in MM express higher PD-L1 vs. MGUS (30). • Lenalidomide + anti-PD-1/PD-L1 inhibits MDSC- mediated immunosuppression (187). • Novel anti-PD-L1 (IgG2a Fc) showed anti-MM activity, possibly via MDSCs reduction (188). • Dara ± anti-PD-1 (cetrelimab) led to decreased CD38^+^ M-MDSCs (189). • Pembrolizumab monotherapy showed some efficacy despite earlier safety concerns (190–192). • LAG-3, TIM-3, and VISTA are also upregulated in MM, supporting broader checkpoint targeting beyond PD-1/PD-L1 (107, 108).	MM patients; MM mouse models; preclinical MM	Supports combining IMiDs/anti-CD38 with CPI to overcome MDSC-mediated resistance.
Synergy with Engineered T-cell therapies	Reducing MDSCs burden to improve CAR-T expansion/function	• High M-MDSCs correlate with poor CAR-T expansion in DLBCL (196). • Anti-CD33 (gemtuzumab) + CAR-T synergistically enhances anti-tumor immunity in solid tumor models (200).	DLBCL patients; solid tumor models(MM validation needed)	Suggests MDSCs depletion (e.g., via CD33) may improve CAR-T efficacy in MM.

PD-1, programmed death 1; PD-L1, programmed death ligand-1; MDSCs, myeloid derived suppressor cells; M-MDSCs, monocytic MDSCs; MM, Multiple myeloma; MGUS, monoclonal gammopathy of undetermined significance; Dara, daratumumab; CPI, checkpoint inhibitor; IMiD, immunomodulatory drug; CAR-T, chimeric antigen receptor T.

Beyond PD-1/PD-L1, other checkpoint molecules such as LAG-3, TIM-3, and VISTA represent promising targets in MM (107, 193). Preclinical evidence indicates that LAG-3 blockade enhances T-cell responses against MM cells (30, 108). However, evidence for VISTA in MM is currently limited to mechanistic and prognostic studies, with no VISTA-targeting agents having entered clinical evaluation (107). Indirect support for broader checkpoint inhibition comes from ARG-1 inhibition, which enhances anti-MM immune responses (preliminary data presented at EHA 2026, Abstract EHA-6462/PS1843) and has shown sensitizing effects to checkpoint blockade in other tumor models (194, 195). Collectively, these findings provide a rationale for combining MDSC-targeting strategies with checkpoint inhibition beyond the PD-1/PD-L1 axis.

#### 5.6.2 Engineered T-cell therapies

Emerging strategies aim to simultaneously target tumor cells and disarm the TME. A prime example is the development of LILRB4-targeting STAR-T cells (an innovative T-cell receptor-based chimeric antigen receptor T cell). This approach demonstrates dual efficacy: direct cytotoxicity against LILRB4-high MM cells and concomitant impairment of MDSCs function, representing a promising integrated strategy to overcome microenvironment-mediated resistance (123). Furthermore, in patients with diffuse large B-cell lymphoma treated with CD19-targeted CAR-T-cell therapy (axi-cel), higher levels of PB M-MDSCs were associated with poor CAR-T-cell expansion (196). Beyond these correlative observations, MDSCs impair CAR-T efficacy through multiple mechanisms, including arginine depletion via ARG-1, secretion of TGF-β and IL-10 that induce T-cell exhaustion, and PD-L1/PD-1 engagement (197, 198). Conversely, CAR-T therapy itself can trigger MDSCs expansion and upregulation of HIF-1α/VEGF/TGF-β signaling, establishing a feedback loop that drives acquired resistance (197, 199). To counteract these effects, MDSCs depletion via anti-CD33 antibody has shown preclinical synergy with CAR-T therapy (200), while engineering CAR-T cells to secrete CD38-targeting bispecific T-cell engagers may simultaneously eliminate tumor cells and CD38^+^ MDSCs, offering a promising strategy to prevent antigen escape and overcome microenvironment-mediated resistance (201) (**Table 5**). These findings provide a strong rationale for exploring similar combination strategies in the treatment of hematologic malignancies.

## 6 Perspectives and future directions

Despite growing appreciation of MDSCs biology in MM, several gaps remain. First, the binary M-MDSCs/PMN-MDSCs classification oversimplifies functional heterogeneity. We propose that single-cell and spatial transcriptomics should be employed to dissect MDSCs subclusters within distinct bone marrow niches.

Second, the optimal combination strategy for MDSC-targeting agents is undefined. Current backbones—bortezomib, lenalidomide, and daratumumab—each partially modulate MDSCs abundance, yet whether MDSC-directed therapies synergize differentially with each is unknown. Our team plans to systematically evaluate, in immunocompetent MM models, the combinatorial efficacy and predictive biomarkers of MDSC-targeting agents paired with these three standard regimens.

Third, we hypothesize that the ultimate potential of MDSCs modulation lies in sensitizing MM to PD-1/PD-L1 checkpoint blockade. The historical disappointment of ICB in MM may partly reflect persistent MDSC-mediated suppression at the time of PD-1 inhibition. We therefore propose that sequential MDSCs depletion prior to ICB—rather than concurrent administration—could reshape the bone marrow milieu and unleash durable T-cell responses. Our planned studies will compare different sequencing schedules to define the optimal timing for such combinations.

## 7 Conclusion

In summary, a bidirectional pro-tumorigenic relationship exists between MDSCs and MM: MDSCs create an immunosuppressive microenvironment that supports MM progression, while myeloma cells in turn recruit and sustain MDSCs populations. MDSCs thus hold dual clinical relevance—serving as potential predictive biomarkers for immunotherapy response and as promising therapeutic targets. Disrupting MDSCs differentiation, function, or surface molecules shows synergistic antitumor potential. Each strategy carries distinct advantages and limitations: depletion effectively reduces tumor burden but lacks selectivity; differentiation offers a physiologic approach with limited clinical evidence in MM; recruitment blockade shows preclinical synergy with immunotherapy yet its MDSC-specific contribution remains unclear; and functional inhibition restores T-cell immunity but faces redundancy and biomarker challenges. However, realizing this potential requires moving beyond empirical combination toward biomarker-guided, rationally sequenced strategies—particularly the optimization of MDSCs depletion timing relative to standard backbones and immune checkpoint blockade. Though further MM-specific validation is needed, targeting MDSCs represents a strategic avenue for enhancing current MM therapies in the precision immunotherapy era.

## Glossary

ARG-1: arginase-1
ATRA: all-trans retinoic acid
BCMA: B-cell maturation antigen
BM: bone marrow
BMSC: bone marrow mesenchymal stem cell
Bregs: regulatory B cells
CAR T: chimeric antigen receptor T
COX2: Cyclooxygenase-2
CSF1R: colony stimulating factor 1 receptor
G-CSF: granulocyte colony-stimulating factor
GM-CSF: granulocyte-macrophage colony-stimulating factor
HDACi: Histone deacetylase inhibitors
HIF 1α: Hypoxia inducible factor 1α
ICB: immune checkpoint blockade
IDO: indoleamine 2,3-dioxygenase
IL-6: Interleukin-6
IL-10: Interleukin-10
IL-18: Interleukin-18
IMiDs: immunomodulatory drugs
iNOS: inducible nitric oxide synthase
LXR: Liver X receptor
M-CSF: macrophage colony-stimulating factor
MDSCs: Myeloid-derived suppressor cells
MGUS: monoclonal gammopathy of undetermined significance
MIF: migration inhibitory factor
MM: Multiple myeloma
M-MDSCs: monocytic MDSCs
NOS2: nitric oxide synthase 2
OS: overall survival
PB: peripheral blood
PD-1: programmed death 1
PD-L1: programmed death ligand-1
PDE5: Phosphodiesterase 5
PGE2: prostaglandin E2
PIs: Proteasome inhibitors
PMN-MDSCs: polymorphonuclear MDSCs
PTGS2: prostaglandin-endoperoxide synthase 2
ROS: reactive oxygen species
RRMM: relapsed/refractory multiple myeloma
SMM: smoldering MM
TAMs: tumor associated macrophages
TGF-β: transforming growth factor-β
TME: tumor microenvironment
Tregs: regulatory T cells
VEGFR2: vascular endothelial growth factor receptor 2
